# Diverting blame to stay sane - young people’s strategies for dealing with the mental health effects of precarious employment: a grounded theory study

**DOI:** 10.1186/s12889-020-08626-4

**Published:** 2020-04-28

**Authors:** Susanna Toivanen, Anna Olofsson Tarantino, Maria Emmelin, Per-Olof Östergren

**Affiliations:** 1 School of Health, Care, and Social Welfare, Mälardalen University, 721 23 Västerås, SE Sweden; 2 Social Medicine and Global Health, Department of Clinical Sciences Malmö, Lund University, 202 13 Malmö, SE Sweden; 3 First Line Clinic, Paediatric Psychiatry (BUP), Skåne University Hospital (SUS), Davidshallsgatan 16, 252 02 Malmö, Sweden

**Keywords:** Qualitative, Grounded theory, Precarious employment, Social capital, Mental health, Youth, Sweden

## Abstract

**Background:**

Precarious employment is a risk factor for poor mental health, particularly among young adults. Knowledge about how young people maintain their mental health while in a precarious employment situation is scarce. The aim of the study was to explore the meaning of precarious employment for young adults in Sweden and their strategies for maintaining good mental health.

**Methods:**

In-depth interviews were conducted with 15 individuals (9 men and 6 women) aged 20–39 years in a precarious employment situation. Contact persons at union offices and at specific job-coaching organizations collaborating with the Swedish public employment agency in the city of Malmö were gate openers to reach informants. Analysis was based on constructivist grounded theory, implying an emergent design where data collection and analysis go hand in hand.

**Results:**

All informants had completed secondary school in Sweden, and one third had studied at the university level. A majority currently had jobs; however, they were mostly employed on an hourly basis and only a few had temporary full-time jobs. The analysis resulted in a core category “Diverting blame to stay sane,” which summarized an emergent coping process involving individual resources and resources represented by the individuals’ social capital. The developed theoretical model contained four main categories, “Facing reality,” “Losing control,” “Adapting,” and “Fighting back,” related to the core category.

**Conclusions:**

The results implied a process where the challenges created by loss of employment-based rights required a coping process where the individual’s social capital plays an important role. However, social capital is to a large extent determined by contextual factors, underlining the strong health equity aspect of precarious employment.

## Background

Mental health is crucial to quality of life and the ability to cope with life’s ups and downs [1] and a key issue on the global public health agenda [2]. Recently, a main concern has been the rising prevalence of mental illness among young individuals with an insecure relation to the labour market [3]. Unemployment, unsafe working conditions, and precarious employment in terms of short-term contracts, involuntary part-time work, and employment through “staff-for-hire” enterprises and online platforms such as Uber are increasingly common among young adults with all levels of educational background [4, 5]. These types of circumstances have proven detrimental to mental health in population-based studies [6–12]. Moreover, precarious employment constitutes an emerging social determinant of health that contributes to health inequalities in working populations [13, 14]. Recent research has demonstrated that young people might be especially vulnerable to health problems when working in precarious conditions [3].

According to our review of the literature, relatively few studies on precarious employment and mental health have focused on individuals in the phase of life when they enter and establish adult life [3, 15, 16]. This time is when they start their working careers; become independent adults with their own dwellings; consolidate their adult near relations, family formation, and other social relations, which all have a major influence on mental well-being. Wyn and Andres [16] reported young people took on average 14 years from leaving secondary school to find stable employment security, and this period was clearly associated with a higher risk of poor mental health, and lower rates of marriage and fertility. In a study from Sweden, precarious employment at two measuring points over a six-year period predicted a 40% higher risk of poor mental health 5 years later among young individuals [15].

In a recent scoping study that included 16 studies of working conditions or precariousness in relation to mental health outcomes among young persons, five studies reported support for the social causation hypothesis that plays a significant role in creating health inequalities [3]. Causal mechanisms explaining these associations included lack of economic and social benefits, exposure to hazardous work environments, a life course perspective in terms of precarious employment being a life event contributing to marginalization, and social exclusion.

Recent studies have linked relations to the labour market, psychosocial working conditions, and different forms of social capital. For instance, social capital at work, defined as norms of reciprocity and trust, was found to protect against poor mental health [17, 18]. The experiences that individuals encounter in working life may thus shape their social capital, depending on which forms of societal bonds are available by means of inclusion in stable work, and the opportunities to control one’s destiny by participation in society through work. In general, research has indicated associations between participation in social networks, trust, and reciprocal norms, namely, social capital and mental health. However, social capital can also in certain situations, e.g. among immigrant women, prevent individuals to get a foothold on the labour market [19].

Young individuals have elaborated on precarious employment and illustrated how control over work and life has been shrinking, resulting in hopelessness and sorrow over not being able to live life according to one’s wishes and society’s expectations (e.g. [20]). In an interview study with adults, Waenerlund [10] and colleagues found that temporary employment was connected to feelings of worry, anxiety, stress, sleeping problems, and risk of developing declining health habits. Because little is known of how young people maintain their mental health while in a precarious employment situation, we conducted a qualitative study among young individuals in the city of Malmoe, Sweden, based on Guy Standing’s [21] investigation of the precariat and the role of social capital as a point of departure. The aim of the study was to explore the meaning of precarious employment for young adults and their strategies for maintaining good mental health.

### Theoretical framework

#### Precarious employment

The British economist Guy Standing [21, 22] has in several articles and books described certain notable effects of the globalized labour market, which do not spare the traditional welfare states, such as Sweden. In summary, global competition has increased the pressure on employers to cut the cost of labour at the same time as mobility of both capital, goods and even the site of production has become greatly increased by means of technical development and deregulation policies. This phenomenon has radically undermined the bargaining position of labour to defend the traditional social contract of the welfare state, where a number of rights have been linked to employment. The cost of those rights for the employer is considerable, making them a prime target of cost cutting, which might be a better option for them than cutting wages, according to Standing. He claims that five types of rights are in focus in this context: Economic rights (e.g., job security, income security, bargaining rights regarding income and employment terms); Social rights (e.g., occupational health and safety and overall social protection); Political rights (e.g., participation in political life and in civil society); Cultural rights (e.g., cultural inclusion and non-discrimination); and Civil rights (e.g., equal rights in judicial contexts and the right to equal treatment and dignity) [22]. Most of these rights are directly or indirectly linked to a secure and long-term employment contract, as an integral part of the social contract that has been established after decades of negotiations between labour and capital in the welfare states, particularly of the so-called Nordic type [23]. In Sweden, this phenomenon has been labelled “the historical compromise” and has led to a situation where the fulfilment of the mentioned rights has become an important share of the cost for labour in the welfare states [24]. However, precarious employment is also on the rise as an integral part of the new global economy, as described in a recent book edited by Neufeind et al. [25].

#### Social capital

Putnam’s [26] definition of social capital as social participation, trust, and reciprocity norms focuses on community characteristics (collective social capital), and others have also viewed social capital as an individual resource (individual social capital) available through their participation in social networks [27]. Social capital studies have largely focused on the potential positive effects of social capital on health, but today social capital is recognized as also encompassing possible risks of exclusion both on community and individual levels [28, 29]. Various studies have indicated positive associations between individual social capital and self-rated health and with mental health [29, 30].

The inspiration for this study is Standings definition of a precarious employment situation and his focus on a rights perspective regarding its possible consequences. Social capital a possible protective factor against the potentially negative health impact of stressors such as unemployment, precarious employment, and job stress. Young people are of particular concern because social capital could have an even more important role in shaping individuals lifetime trajectories of mental health, or shaping a potentially life-long resource of importance for maintaining good mental health. We use these concepts to guide our data collection and analysis, and so-called *sensitizing concepts* help us to find directions regarding what type of information to search for, but this method did not limit the scope of our observations [31].

## Methods

### Overall study design

To capture the experiences of the informants, the study was based on qualitative methodology [32]. We referred to Charmaz [33] constructivist grounded theory because we specifically aimed to understand what it means for young people to be in a precarious employment situation and what strategies they use to maintain good mental health. Similar to Charmaz, we consider grounded theory as constructed in the interaction between the researcher and informants, producing a “portrayal of the studied world, not an exact picture of it” [33].

### The setting

The study was performed in Malmö, the third-largest city in Sweden with approximately 320.000 inhabitants. Nearly half the population is younger than 35 years of age, and 30% of the youth has at least secondary education [34]. One third of the population is born outside Sweden, and the largest of those groups are from Iraq and Denmark. Malmö has a broad variation of industries; notably, 70% of work opportunities are within the private sector and 30% in the public sector. The unemployment rate was 15% at the time of the study, which is double compared with Sweden as a whole, and for youth aged 18–24 years, it was as high as 23%. However, large differences in unemployment between men and women, foreign and Swedish born, and youth and others are observed.

The Malmö Commission [35] describes how Malmö has been affected by the global trend of moving away from traditional employment based on collective bargaining and union organization. New types of precarious work offered as project employment, internships, and staff-for-hire enterprises imply specific difficulties for youth, migrants, and others who have a weakened connection to the labour market. Stigendal [36] pictures Malmö as a deeply polarized and segregated city as a result of harsher labour market conditions and increased income inequality. Social position and ethnicity largely determine where people live. Presently a majority of the population lives in resource-poor settings with a high proportion of unemployment. Despite these inequalities and marginalization, Malmö is also portrayed as a cosmopolitan centre with cultural, educational, and innovation opportunities.

### Sampling of informants

The sampling strategy was purposive. We aimed to reach young adults aged 18–35 years working in branches characterized by offering precarious job opportunities in the Malmö area. This type of work was found in many branches, but we decided to focus on employment in hotels and restaurants, departmental stores, transport services, social and healthcare sectors, and academia. We wanted to reach maximum variation in experiences of precarious employment and included young men and women from varying educational, socioeconomic, and ethnic backgrounds.

To recruit informants, we approached the union offices in Malmö and specific job-coaching organizations by collaborating with the Swedish public employment agency. Our contact persons acted as gate openers by approaching individuals that met our sampling criteria and informed them briefly about the study. If interested, potential informants agreed that the gate opener could provide their contact information to the research team. The interviewer could then contact them to suggest a time and place for an interview.

### Data collection

The second author (AOT) performed all interviews between September 2015 and April 2016. Fifteen interviews were conducted, of which 14 were individual interviews. One interview included two persons from the same workplace who requested to be interviewed together. Prior to the interviews, the informants were provided information about the aim of the study and the opportunity to ask questions before giving their consent. The interviews were held in different locations, depending on the preference of the informants. Seven informants were interviewed in a prearranged office at the interviewer’s workplace, three were held at one of the coaching centres, two at the informants’ workplace, two in cafe’s in Malmö, and one in a private space at Malmö University. Except for the interviews in cafe’s, a quiet place with little disturbance was provided.

An interview guide was developed for the present study (Additional file). The interview guide was tested in a pilot interview, and the revised version included questions on background information in terms of education and family situation as an introduction to more-detailed questions on social network, work life experiences, economic situation, reflections on what precarious employment entails, and mental health. Basically, the informants were asked to tell their story in relation to their work experiences so that the interviewer could probe on these areas. The duration of the interviews was between 55 and 80 min, and all were digitally recorded after consent from the informants.

### Analysis

The analysis was based on constructivist grounded theory in Charmaz [33], where a preliminary analysis is performed along with the data collection. Thus, after each interview, the tapes were listened to, and an *initial coding* process was started. Memos were written on a regular basis to capture ideas about the codes that were notable. These memos were constantly revised and expanded to also cover data from forthcoming interviews. This method provided the opportunity for comparison and for sharpening ideas that occurred and could be elaborated in the next step of a more *focused coding*. Discussions regarding the findings in the interviews also were held during the process of writing memos.

**Fig. 1:**
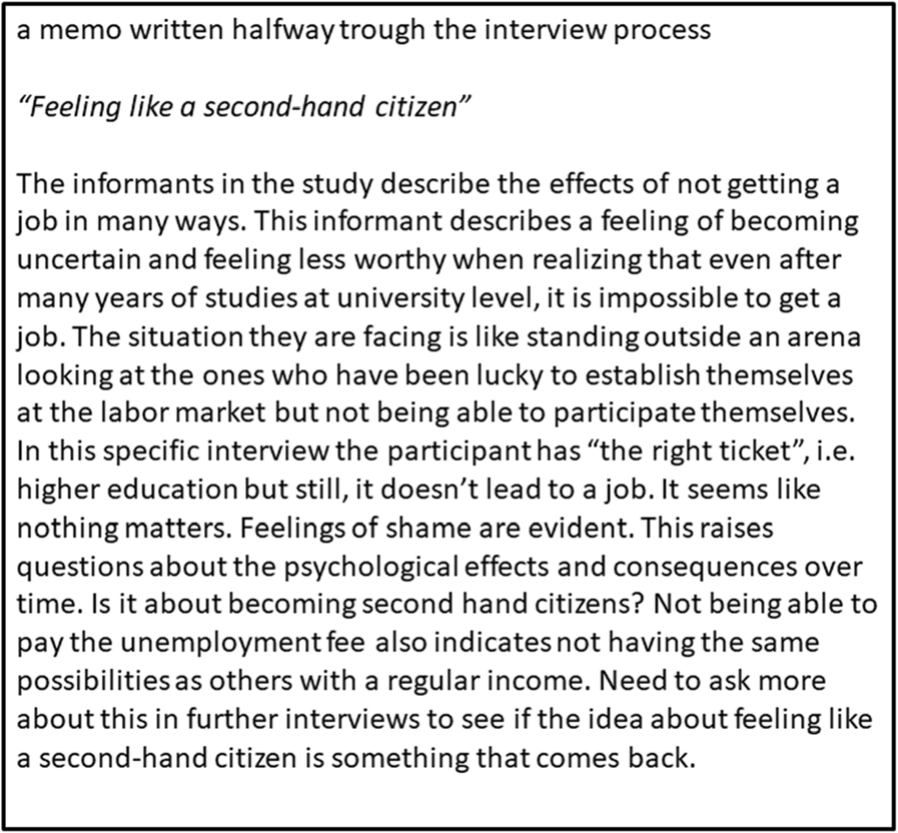
An example of a memo describing one of the emerging sub-categories

During and after the focused coding, tentative categories were constructed to represent the meaning of precarious employment and the strategies used to maintain mental health. Memos played an important role in saturating the categories to cover their properties and dimensions. By going back and forth between text, codes, and categories, new categories were constructed to cater to upcoming ideas. After eight interviews, the categories started to become saturated, but we decided to continue to sample more informants to feel assured that we had not missed important variations in our data. After 15 interviews, no new ideas emerged, and we concluded data collection. The final analysis continued by re-reading the interviews and the memos to negotiate the interpretation in peer-debriefing sessions within the research group and to revise the codes and re-label the tentative categories. Figure 1 provides an example of a memo written during the analysis process on one of the final sub-categories.

## Results

Of the 15 informants, both men and women, all except one were within the initial target group of 18–35 years. Their age ranged from 20 to 39 with a mean age of 27 years. Because the informant aged above 35 years had long experience of having a precarious employment situation, we included this interview in the analysis. Some socio-demographic characteristics are given in Table 1. Most of the informants were born in Sweden, and the other informants represented countries within and outside Europe. All informants had completed secondary school in Sweden, and one third had studied at the university level. A majority currently had jobs; however, most were employed on an hourly basis, and only a few had temporary full-time jobs. They had experiences from a broad range of branches such as hotel and restaurant, transport and logistics, trade, elderly care, school and education, and financial services. However, most of the employed informants currently worked in branches not related to their educational background.

**Table 1:** Socio-demographic characteristics of informants

Sex	Country of origin	Education in Sweden	Work	Sector
Woman	Sweden	Secondary school	Hourly	Public
Woman	Sweden	Secondary school	Hourly	Private
Woman	Sweden	Secondary school	Unemployed	–
Woman	Outside Sweden	Secondary school	Unemployed	–
Woman	Sweden	Secondary school	Temporary/Full time	Private
Woman	Sweden	Secondary school	Hourly	Private
Woman	Sweden	University	Hourly	Public
Woman	Sweden	Secondary school	Hourly	Public
Woman	Sweden	University	University studies	–
Man	Outside Sweden	University	Hourly	Public
Man	Outside Sweden	Secondary school	Hourly	Private
Man	Outside Sweden	University	Temporary/Full time	Private
Man	Sweden	Secondary school	Hourly	Private
Man	Sweden	University	Hourly	Private
Man	Outside Sweden	Secondary school	Hourly	Private

### Diverting blame to stay sane

The analysis resulted in the construction of one core category “Diverting blame to stay sane,” which was interpreted as a means for young people to cope with or overcome the mental stress that being in a precarious employment situation meant to them. However, we clearly observed from the analysis that the direction of blame varied from taking on full individual responsibility for their precarious situation to fully placing the responsibility on the societal and structural contexts, namely, the labour market and the lack of opportunities provided.

**Fig. 2:**
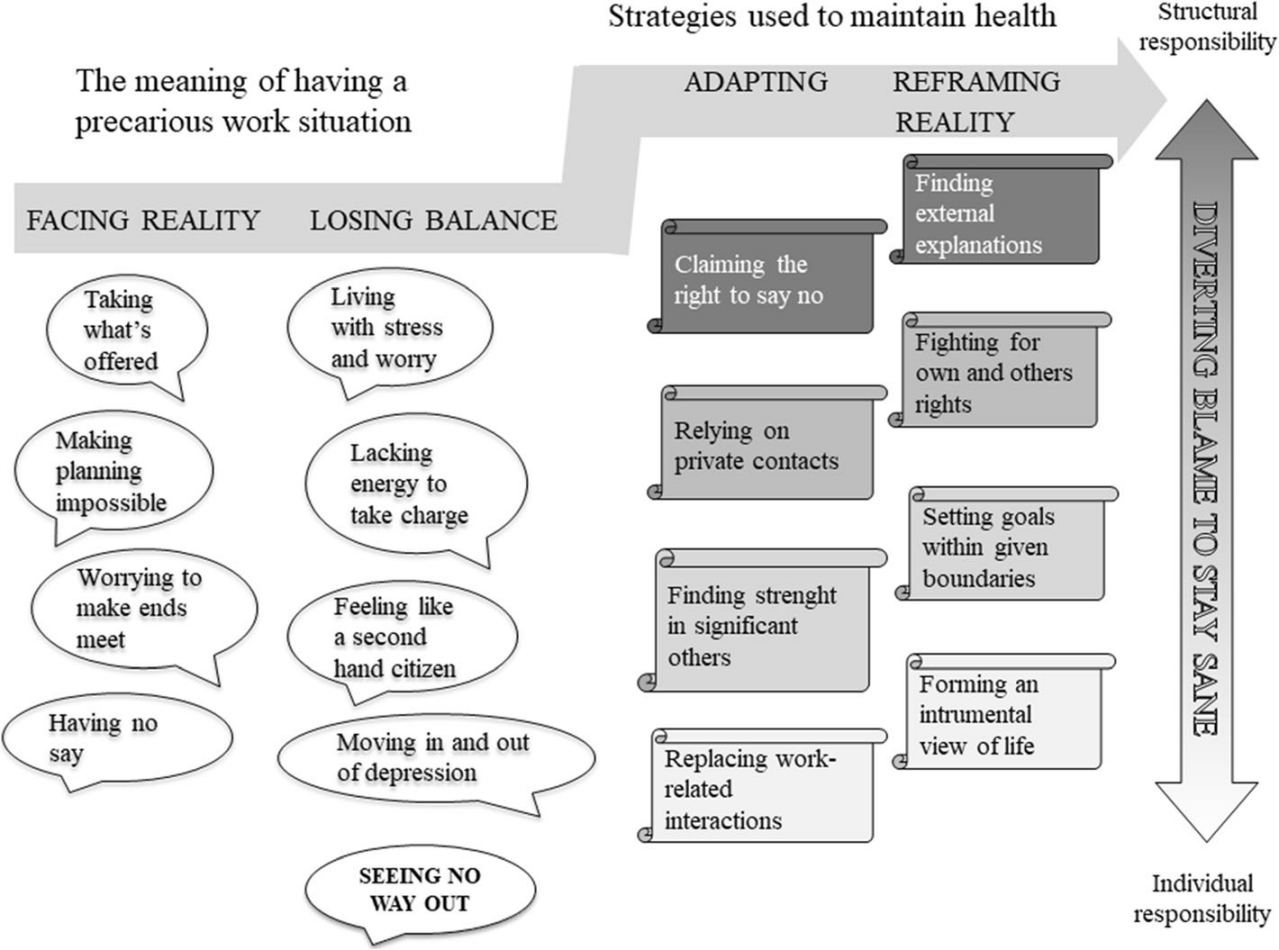
A theoretical model of the links between the core category “Diverting blame to stay sane” and the main categories “Facing reality,” “Losing balance,” “Adapting,” “Reframing reality,” and their properties

The hypothesis we proposed is as follows: the more young people manage to avoid taking on individual blame, the less likely the precarious employment situation is to influence their health negatively. Figure 2 provides an overview of the developed theoretical model with the four main categories, “Facing reality,”” Losing control,” “Adapting,” and “Fighting back,” and all are related to the core category. The first two main categories focus on what precarious employment means to them, the last two points more directly to the strategies used to maintain health. The sub-categories show variation in experiences, re-actions, and strategies during the process that to a varying degree influence their health. In Fig. 2, these sub-categories are placed in relation to individual and structural responsibility to depict what re-actions and strategies are protective and what represents threats to health. In the more detailed presentation, the results are presented under the headings of the main categories with the sub-categories in bold italics in the text indicating their properties. Quotes are provided to support how the interpretation is grounded in the data.

### Facing reality

The category facing reality comprised aspects of what it meant for young people to realize the limited job opportunities available. In retrospect, they could blame themselves or others for having made wrong decisions in high school by not thinking about the implications on job opportunities. Others felt they made the right choice but still could not find a job as they entered the labour market. Facing reality meant realizing that they had to accept the jobs offered and that there was no chance of receiving a full-time job, only part-time or hourly job. Reality was perceived as difficult and merciless, resulting in social and psychological consequences that affected their self-confidence negatively.

The informants’ narratives indicated a unanimous picture of frustration with their work situation resulting from having *to****take what was offered***— or not get a job at all. None had actually been able to obtain jobs of the type they wanted or were educated for. Often they had started with an internship, a type of job that often was extended repeatedly but rarely led to a permanent job. The job opportunities offered varied from having to be in standby mode all the time, just in case the employer would offer them a job for the day, to having a fairly regular schedule to follow but still on a temporary basis. The expectation to be grateful for getting a job, even if only temporary, was sometimes felt as a burden.

*"It sounds good. I mean you have a job, they call and you get money. But how long will they keep calling? How many times do I have to sacrifice my free days?”* (Interview 4, man)

The informants described how their work situation ***made planning impossible*** because they did not know if they would need to be on standby waiting for phone calls or an SMS. This situation affected their social life in relation to partners, children, families, and friends, even if they and their loved ones tended to become accustomed to this inconsistency. For those with children, being unable to plan became extra problematic. Having to leave children at day-care early in the morning, just in case there would be work that day caused guilt about being a bad parent. Not being able to plan also influenced their more long-term planning for adulthood by, for example, limiting their possibilities to enter the housing market. They were unable to obtain a rental contract and could not obtain bank loans without contacts or relatives prepared to become their creditors or provide private loans.

*“It makes life difficult, you get stuck for example with housing … you can’t apply for any housing because you can’t prove that you earn money, even if you have actually earned money and paid your rent for 10 years...”* (Interview 1, man)

A precarious employment situation also implied a constant ***worry about making ends meet***, namely, never knowing how much money would be left at the end of the month. Some months individuals would earn a large salary, but in other months, that salary could be reduced to nearly nothing, generating a lot of stress. Temporary employment or being hired on an hourly basis also meant not obtain the salary until the month after, which took time to become accustomed to. For women in relationships and with children, this variation in salary could cause tension and force them into stereotypical relationships where they would be homemakers the man compensated for the one income by working more and longer hours. Many narratives illustrated how worries about irregular income resulted in not being able to buy even the most necessary things and how it limited their social interaction with friends.

*” … can I buy new clothes this month or do I have to wait until next month? When friends call and ask if you want to go out with them, you have to think, ok, how much is this going to cost? … and then if you do decide to go out with them you have to think, where can I save money in these next couple of weeks so that I can actually afford this …*” (Interview 13, woman)

This worry also made parents feel guilty for having to deny their children leisure time activities such as going to the movies. From the interviews, we clearly observed that being hired temporarily or hourly meant ***having no say*** about the working conditions and made it difficult to have demands or make complaints. Some described how this made them feel exploited and controlled by the employers, still understanding their standpoints, and thus facing reality.

### Losing balance

This category illustrated how being in a precarious employment situation manifests, to different degrees, by making young people lose balance in different ways. The joint experience of living with stress and worry takes its toll and makes it hard to believe in your capacity and your possibilities to influence the situation. The effects on mental health vary and range from tiredness and sleeping problems to diagnosed depression and losing self-confidence and faith in possibilities for improvement.

For the informants, not knowing how their employment situation would develop resulted in ***living with*constant*stress and worry*** and manifested in sleep disturbances that influenced their daily life.

*” There haven’t been many nights I have had my full 8 h sleep … You just get a bit worried that’s all, like there is always something in the background, which is worrying me, then you realize that is must be work. Then you come here (to work) and feel calm because everyone is so supportive, saying that everything is going to be ok, you won’t be without a job with your experience … but as soon as you leave here, the uncertainty is back again.”* (Interview 5, woman)

Sleeping problems were also related to unhealthy eating, and excessive alcohol consumption was described as one means to cope with a stressful work situation. The experience of having sought emergency care from mistaking the symptoms of stress for severe illness was recalled in several of the narratives. The informants could also clearly describe the vicious circle that living with stress and worry resulted in. The level of stress was high almost all the time because they never knew for sure if they would be offered a work opportunity and also knew that if they turned down an offer that that could result in no more offers. Not knowing could also cause competition and irritation within a relationship.

Stress could also result in feeling powerlessness and blaming themselves for not having a stable job situation even if intellectually understanding that this was not the case.

*” The stress makes me feel powerless and I can get very upset. I feel like it’s my fault that I should be doing something about it and that really smart people don’t end up here … like it really affects your confidence a lot. Even though, at the same time, I know intellectually that no, that’s not how it is actually.”* (Interview 8, woman)

The informants’ stories clearly indicated that being in a precarious employment situation for a longer time resulted in ***lacking energy to take charge***, and they described becoming increasingly passive. After having too many work applications rejected, they started to feel that searching for a job was a pointless struggle and started to consider other solutions for making money. Others attempted to keep their hope up but described the short distance between losing faith and giving up.

The informants portrayed being in a precarious employment situation as ***feeling like a second-hand citizens***, that is, not having the same rights or opportunities as others. Constantly having to be in a standby mode, not being able to say no, and the constant worry about money were the most prominent concerns making them feel shame and less worthy as human beings. Additionally, the limited possibility of having a decent home was perceived as socially degrading and was pointed out by informants with and without children. The following narrative illustrates what it meant to one informant to not be able to choose where to live and fulfil her dreams.

*“Now we live in like a slum … I would not have said no to living in an area where my children could be outside and play on their own and I could see them from the window. I can do that now too, but there are needles out there, there are young people who use drugs and such, I don’t even go out there on my own with my children … So you check out you know, what it would be like if we lived there with that kind of life. But then you look at the rent and say, no, we have to stay in the slum a while longer … so you just have to dream for now … I think about these things a lot at the moment …*” (Interview 6, woman)

Based on the informants’ narratives, the joint experience of living with work-related stress and worry increased the risk of ***moving in and out of depression*** or depressive related symptoms.

*” It’s awful, I have burnt-out many times. My husband has also had a burnout, he was on sick leave for 6 months because of stress, before he’d even turned 30 …*” (Interview 7, woman)

However, the difficulty of recognizing or acknowledging depressive symptoms was observed in that they were described by the informants merely in terms of tiredness and passivity. Not being able to recognize depression could also result in food self-medication with consequences for other aspects of health. Being a parent could make becoming depressive because easier to admit because of the obvious consequences on the ability to care for children. The following quote illustrates how one of the informants cried out for help but experienced how difficulty it was for people around them to recognize what was occurring.

*”... I mean I noticed that I just wanted to sleep and that I couldn’t even get myself out of bed. Everyone went around and asked why I only slept, what was wrong with me and everyone went around complaining about how I looked. I have only lost a bit of weight and now everyone is asking me what I eat and things like that. I was completely shocked, I mean what is this? It is not that I don’t want to eat, I mean I do eat it is just so … don’t you see that I have no energy left?”* (Interview 9, woman)

The informants who had experienced not being able to obtain a job for the longest time and/or having a criminal record expressed the most hopelessness*,****seeing no way out*.** Having exhausted the available possibilities, pursued an education, and applied for job after job and still not having gotten a job opportunity was perceived as devastating. For parents, there was an additional concern about the consequences for the coming generation of not being able to be a good parent without a regular job.

### Adapting

This category points to different strategies used to adapt to the situation and thereby counteract the negative impact that the precarious employment situation had on health and social interactions. How this was accomplished differed and included actively opposing the situation by not accepting everything offered and trying out new methods to obtain jobs. It also meant exploring alternative opportunities for meaningful interaction with other people but also to depending heavily on the availability of family and significant others.

One meant to adapt at least partly to the situation was ***claiming the right to say no*** to job offers that did not feel suitable. This action increased the informants feeling of being autonomous and able to make decisions over their own lives, but the opportunity seemed mainly available for those with higher education and aspirations.

*” I am actually a qualified economist, on that level, but I have even considered applying for jobs as an economist’s assistant which are lower just to try and get a foot in. But I haven’t taken jobs as a cleaner … or jobs which aren’t related to what I do …*” (Interview 2, man)

Another argument for the right to say no was to refer to health reasons to claim that they could not take a job that would make them sick in the long run. However, we also observed that the choice of saying no to job offers was not an easy choice and could be met by resistance from family and friends.

***Relying on private contacts*** and avoiding public institutions such as the public job placement service to obtain job offers was an important lesson learned. Others described how they, even for temporary employment, depended on knowing to the employer and that only by having these contacts would they even have an opportunity to work.

*” I worked for example at X and that was just because there were many people at my other job who also worked there … I mean that’s how it works, that’s how you get work.”* (Interview 1, man)

From the interviews, we clearly observed that having the possibility of ***finding strength in significant others*** such as a caring family or children to take care of made a big difference in their ability to cope with the consequences of being in a precarious employment situation. The respondents with supportive family or friends described how they helped them keep up their motivation and hope when feeling low after too many rejections of job applications.

*” … I have friends and relatives who tell me, don’t give up it is going to happen sometime, I can help you, we can go out and … you can come over to my place and we can apply for some jobs together, I have always had people who push me...”* (Interview 10, woman)

Having a combination of supportive friends and relatives that offer practical help and the responsibility for a pet animal was also mentioned as increasing the possibility of maintaining mental strength. For parents, having children significantly motivated them to continue striving to provide their children a decent childhood. Children provided energy when times were tough.

The informants also described how being in a precarious employment situation forced them to find new arenas to meet people to ***replace work-related interactions*.** The internet had obviously become a critical means of communicating with others and gaining new friendships when not involved in workplace relationships.

*” I mean we live in a world where there is so much choice. I spend a lot of time online with my fellow bloggers and even though I want to shut down my blog I haven’t dared to because what happens when I don’t have that community?”* (Interview 7, woman)

To maintain their mood and to avoid social isolation, others described their efforts to continue to go out with friends or have friends visit, despite having no money to spend. They struggled to find activities that were free or low cost; then, they could meet with their friends outside their home. There were also informants who emphasized the importance of physical exercise and described how this activity became a means to avoid being idle and made them feel happier about life.

### Reframing reality

This category refers to the different explanations of precarious employment and how the informants started to reframe their reality to be able to maintain health. The explanations included finding different structural explanations to distance themselves from responsibility to merely accept the situation and hope for better times.

Among those from a working-class background, **finding structural explanations** was not far-fetched regarding their present work situation based on their limited resources during childhood.

Others were from a more middle-class background that reflected in a similar way and pointed out how they were indeed privileged and maintained hope about their prospects. For a person with a criminal record, this situation was an evident explanation for the experiences of closed doors to the labour market but also seemed to help with understanding the situations for those without.

*” In my case, I thought instantly that it’s something to do with my record, so it’s to be expected. But for other people who have a clean record and education and they apply for jobs over and over, of course it affects them. I think they feel a lot worse than I have done.”* (Interview 4, man)

The recent influx of refugees was also used as a partial explanation for their difficulties in the labour market, illustrating a harsher societal climate where vulnerable groups are put against each other.

*” Ok, now I am going to sound really racist, but I am not very happy with all these new refugees who have got ‘start-up jobs’ … What about me? Should I not also get help with a start-up job for example? I am a little envious of them. I feel that Sweden is more interested in helping them … Sweden is more interested in helping everyone apart than their own.”* (Interview 10, woman)

An important driver to take some form of action and **engage in own and others rights** was the anger and frustration that being in a precarious employment situation had created. For some of the informants, this led to joining a union, which also helped them maintain their health.

*” Of course it affects me, because I am quite angry about how we are being treated. I have also started getting involved with the unions, it is a driving force which makes me feel better, I think … I am trying to make a difference, but it also just makes me even angrier.”* (Interview 1. man)

Engaging in creating a common good was also regarded as making it possible to get through the day and see the work as meaningful because of the efforts to improve work conditions for others. Being involved in fighting for theirs and others rights also resulted in contacts with people with similar values and contributed to new friendships. For others, it was more of a general stance, that is, not accepting their situation and wanting to get more out of life by doing good.

*” And I think about that all the time, what is the meaning of life? I feel like I am really not cut out to be a criminal or to work for a recruitment agency or any of that … there are other things that a person should do with their lives and that is what I have tried to do and that is what has made sure I haven’t given up or gone under...”* (Interview 4, man)

***Setting new goals within given boundaries*** was another strategy that the informants described as developing over time and helped them stay hopeful and feel that they mastered the situation. For example, turning experiences of a temporary job into something meaningful that they could use further on and that they felt grateful for. There were also informants that after being unemployed for a long time perceived that this was a time that had to be endured. Even if continuously failing to get a job felt like being in a roller coaster, the only way forward was to look positively at the future and try to identify new means of finding a job.

*” I think the first year is really important … it’s just something you have to go through, and I think that’s when you realize … it’s like a roller coaster you’re on. I would probably say that the first year and half were not so positive … but you have to realize that yourself … I am much more positive now towards everything and try not to take things too seriously. Yes, you can get through it, but you just have to do things differently sometimes.”* (Interview 11, woman)

In the interviews, we also observed that some informants attempted to distance themselves from the negative thoughts and feelings connected to being in a precarious employment situation in a manner that could be labelled as **forming an instrumental view of life.** Talking about the precarious employment situation was conducted with a mental distance that became a safety net for not becoming desperate but still longing and dreaming about something more meaningful.

*“Work for me at the moment just means that I can pay my rent and buy food. But I wish it maybe meant something else, that it was something that felt meaningful. But that’s not how it is today … I will try and do something about that sometime …* “*(*Interview 1, man)

## Discussion

The results from this study indicate that the process that young people undergo when being in a precarious employment situation can be interpreted as **diverting blame to stay sane**. Those that manage to avoid blaming mainly themselves were observed to be less affected by the constant stress of precarious employment and thus managed to stay healthier. The process starts with **facing reality** and illustrating their joint meaning of precarious employment, characterized lack of influence and worry for the future. These factors lead to **losing balance** due to constant feelings of stress and worry and feelings of being downgraded as human beings with both immediate and more long-term mental health consequences. The strategies used to maintain health were **adapting** to and **reframing reality.** These being in a precarious employment situation away from themselves, giving them a better chance to remain healthy.

Research on individuals entering working life has shown a strong link between precarious work in terms of temporary employment and increasing mental ill-health over time, developing declining health habits [9, 10, 15], and declining opportunities to recreational activities or winding down due to inadequate sleep [37, 38]. There are very clear indications that young adults’ mental health is decreasing in high-income countries [3], and changing labour market relations are critical in this context because they are linked to income security, social relations, access to decent housing, and other factors critical to successful adaptation to adult life [3, 5]. Stable employment partly determines access to social capital, which is essential for handling major demands of adult life. However, there are also reports that social capital could, by means of social obligations, become an obstacle for entering the labour market, especially among immigrant women [19]. In our interviews such situations were not reported.

The qualitative analysis of this study uncovered a coping process over time, which can be interpreted by using Standings [21, 22] definition of precariousness and the concept of social capital [26–30]. Standings [22] discussion of lost rights could be used as a framework to understand a low level of, or even successive loss of, coping recourses in relation to demands and the conceptual framework of social capital as a recourse for compensating for the imbalance between demands and resources. The theoretical model developed from the analysis illustrates different stages in individuals’ coping process: initially facing reality and making a first appraisal of the aspects of precariousness, then experiencing stress because of an inability to cope with the demands, followed by using social capital resources to cope by taking action or redefining the situation and finally the development of new strategies to” fight back” and preserve or regain mental health. The model implies that an important element for the success of this process is whether the individual manages to divert the sense of blame from themselves to external circumstances. This also illustrates awareness of social structures in working life where the responsibility for decent work is shifting from employers to employees, particularly to those in a precarious employment situation [5].

In the quotes from the informants, we observed several examples of the loss of the rights that Standing mentions: Economic rights (e.g., lack of income security, employment security, access to credit); Social, Political, and Cultural rights (e.g., poor access to social protection like unemployment benefits, health insurance, and loss of opportunities for social participation in a broad sense with the risk of social isolation and marginalization); and Civil rights (e.g., the right to equal treatment and dignity). The experience of the loss of the aforementioned rights automatically entails loss of important resources for coping with day-to day challenges, which leads to stress, worry, exhaustion, and low mood/depression. These phenomena are typical initial stages of a potentially long-term decline in mental health. Therefore, the individuals’ social resources, represented by their social capital, can be of decisive importance for their future mental health trajectory.

Notably, as aforementioned, social capital is not only an individual resource but is determined by contextual factors, such as mechanisms of social stratification; moreover, tendencies of marginalization involve residential/housing aspects, cultural/ethnic relations, existence or non-existence of compensatory welfare policies, and civil society factors. The enumeration of these contextual circumstances shows a clear connection with the loss of the individual rights mentioned by Standing and the collective recourses reflected by social capital. Thus, social capital could protect from the negative impact of a precarious employment situation in the short perspective. Yet, in a longer perspective, the same factors are likely to increase precariousness in the labour market and erode individual rights and will in parallel erode collective resources through negative effects on the aforementioned contextual determinants. In such a case, the negative impact of precarious employment on mental health among young individuals might become even more deleterious in a future perspective.

Policy response to the growth of precarious employment seems to have been slow in Sweden and the EU, probably because it represents a “wicked” problem, linked to many societal areas, such as labour market regulation, migration policy, work and safety policy, social welfare policy, and regulation of the use of new technology and of financial markets. However, stressing the negative effect of precarious employment on mental health among an increasing proportion of the workforce may be helpful for coordinating much needed policy, preferably linked to the overarching concept of sustainable development which in fact has evolved for responding to this type of challenges,

Also notable is that diverting blame from oneself to external factors and” fighting back” entails a potentially political mobilization, predicted by Standing [21] in the notion of the precariat as the” new dangerous class”. However, with the important comment that the character of political action might be unpredictable and determined by contextual circumstances such as place, time and prevailing political climate.

All in all, the observations from this study might lend contemporary relevance to the statement by Rudolf Virchow in 1848:” Politics is nothing but medicine at a larger scale” [39].

### Methodological considerations

Both our and others’ previous research have shown that precarious employment is a significant risk factor of mental ill-health among young adults. The aim of this study was to capture how this phenomenon manifests in young people’s life and the strategies used to maintain mental health. A strength of the study is that we theoretically represented the target population by purposively sampling young men and women with different educational backgrounds from different branches of the labour market that were in a current precarious employment situation. A challenge was, however, to reach male informants and persons with ethnic background other than Swedish willing to share their experiences. Thus, our analysis focused more on joint experiences than on differences based on gender and ethnicity. Additionally, the informants were from an urban area with high competition for work opportunities, and that limits the transferability to other settings. To further enhance the credibility of the study, the sampling strategy and the data collection process were closely followed, discussed, and reflected upon within the research group. The second author’s continuous memo-writing played an important role in the development of the main categories and their sub-categories. To increase the resonance of the study, we continued sampling informants after having developed the main categories to ensure that that we had not missed important aspects of their experiences. The originality of the results is supported by the focus on the variation in young adults’ agency in maintaining mental health, rather than considering them victims of circumstances. Thus, the findings are useful both for understanding the distressing health consequences of having a precarious employment situation during early adulthood and for recognizing the support necessary for them to avoid taking on individual blame.

## Conclusions

This study increased the understanding of precarious employment and its effects on young people’s mental health. The results indicate the importance of providing safe employment conditions in this critical life phase when the platform for adult life is constructed. The results further highlight the importance of designing health policies that guarantee fair working conditions across different life stages including the important years of early adulthood. Further research on the theoretical conceptualization of the mechanisms linking precariousness and mental health is encouraged.

## Supplementary information

**Additional file 1.** Interview guide.

## Data Availability

The datasets generated and/or analysed during the current study are not publicly available due to participant confidentiality but are available from the corresponding author on reasonable request.
