# Supplementary material for: Diverting blame to stay sane - young people’s strategies for dealing with the mental health effects of precarious employment: a grounded theory study
Source: BMC Public Health. 2020 Apr 28;20:571. doi: 10.1186/s12889-020-08626-4 (PMC7189722; doi:10.1186/s12889-020-08626-4)
Supplement: Supplementary file 1 — Additional file 1. Interview guide. [file 12889_2020_8626_MOESM1_ESM.docx]

# Interview guide for the study on young people's strategies for dealing with the mental health effects of precarious employment

Susanna Toivanen, Anna Olofsson Tarantino, Maria Emmelin & Per-Olof Östergren

## Background

Tell us a little about yourself…

- Where were you born?
- How have you lived, grown up?
- What did you think of school?
- What is your highest completed education?
- How would you describe yourself as a person?
- What does your work situation look like today?

## Experience of uncertain working conditions

The first time you were unemployed or had a hard time finding a job or jumped between different jobs with precarious employment conditions, tell me what it was like:

- What happened?
- What did you do?
- How did it feel?
- What did you think?
- What felt worst?

During this period:

- What made you get up in the morning?
- How did you spend your days?
- Tell me about a time that felt extra tough?
- Tell me if you felt it was going well, if something was good with the situation?

## Mental health

- How would you describe your mood during this time?
- How did this time affect your mental health?
- Can you say something about how you were affected by being in this work situation?
- What was toughest during this period?
- What feelings did you have?

## Support

- Did you get any help during this time?
- Family? Friends? Network? Social forums? Employment Service? Something else?
- In what way was this a help for you?
- How did you move on?
- How do you react during pressured periods? What do you usually think? What are your strategies for coping with tough experiences?
- Do you have any traits that helped you during this time?
- What could have been different during this period that would have made it feel easier?
- If you could wish for something during this time, what would it have been? How had it been?
- What is the most important lesson learnt from this time?
- What advice would you give to another person who is in the same situation?
- If you were to describe this time in three words, which words would you use?

## Current state

Tell me about your life right now:

- How would you describe your mental mood/health today?
- Your physical health?
- Has your mental mood/health changed? What has led to this change?
- If you were to compare your mental mood/health then and now, what's the difference?
- Have your experiences changed you in any way? How?
- If your mental mood/health was negatively affected during this period, what made you get to where you are today?

## Reflections on the interview

- Is there something important that we have not touched upon, something we’ve missed?
- Is there anything else you think I should understand better?
- Anything you would like to change or clarify?
- Do you have any questions for me?
- May I summarize a little how I have understood what you have said, then we can see if you think I have understood you correctly?
